# Supplementary material for: Spatial-temporal regulation of the prostanoid receptor EP2 co-ordinates PGE2-mediated cAMP signaling in decidualizing human endometrium
Source: iScience. 2024 Oct 16;27(11):111170. doi: 10.1016/j.isci.2024.111170 (PMC11567134; doi:10.1016/j.isci.2024.111170)
Supplement: Document S1. Figures S1–S3 and Tables S1–S5 [file mmc1.pdf]

iScience, Volume *27*

Supplemental information

Spatial-temporal regulation of the prostanoid receptor EP2 co-ordinates PGE2-mediated cAMP signaling in decidualizing human endometrium

Paul J. Brighton, Abigail R. Walker, Oliver Mann, Chow-Seng Kong, Emma S. Lucas, Pavle Vrljicak, Jan J. Brosens, and Aylin C. Hanyaloglu

Supplemental Information

Spatial-temporal regulation of the prostanoid receptor EP2 co-ordinates PGE2-mediated cAMP signaling in decidualizing human endometrium

Paul J. Brighton, Abigail R. Walker, Oliver Mann, Chow Seng-Kong, Emma S. Lucas, Pavle Vrljicak, Jan J. Brosens and Aylin C. Hanyaloglu

Figure S1

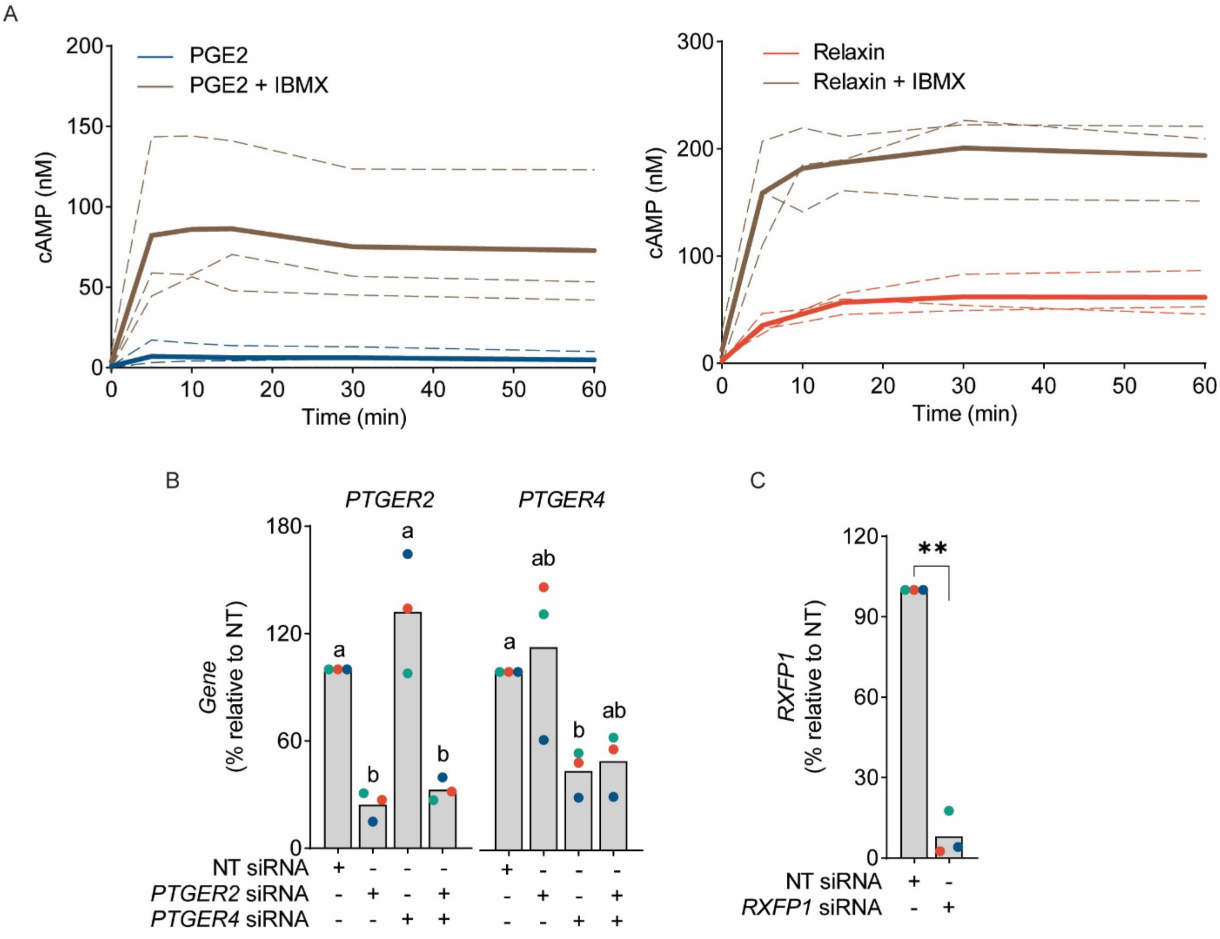

Figure S1. Characterization of cAMP signaling by PGE2 and Relaxin in EnSC, related to Figure 1. (A) Changes in intracellular cAMP induced by PGE2 or relaxin in EnSCs in the presence or absence of the phosphodiesterase inhibitor, IBMX. Plots from individual patients are represented by dashed lines with bold lines indicating mean values, n=3. (B) siRNA-mediated depletion of *PTGER2* and/or *PTGER4* in EnSC, as confirmed by RT-qPCR. Data are shown individual biological replicates from independent primary cultures with bars denoting mean values. Different letters indicate statistical difference ($p < 0.05$) from NT siRNA, stimulated cells (ANOVA and Dunnett's multiple comparison test). C) siRNA-mediated depletion of *RXFP1* in EnSC, as confirmed by RT-qPCR. ** indicates $p < 0.001$ from Student's t-test, n=3.

Figure S2

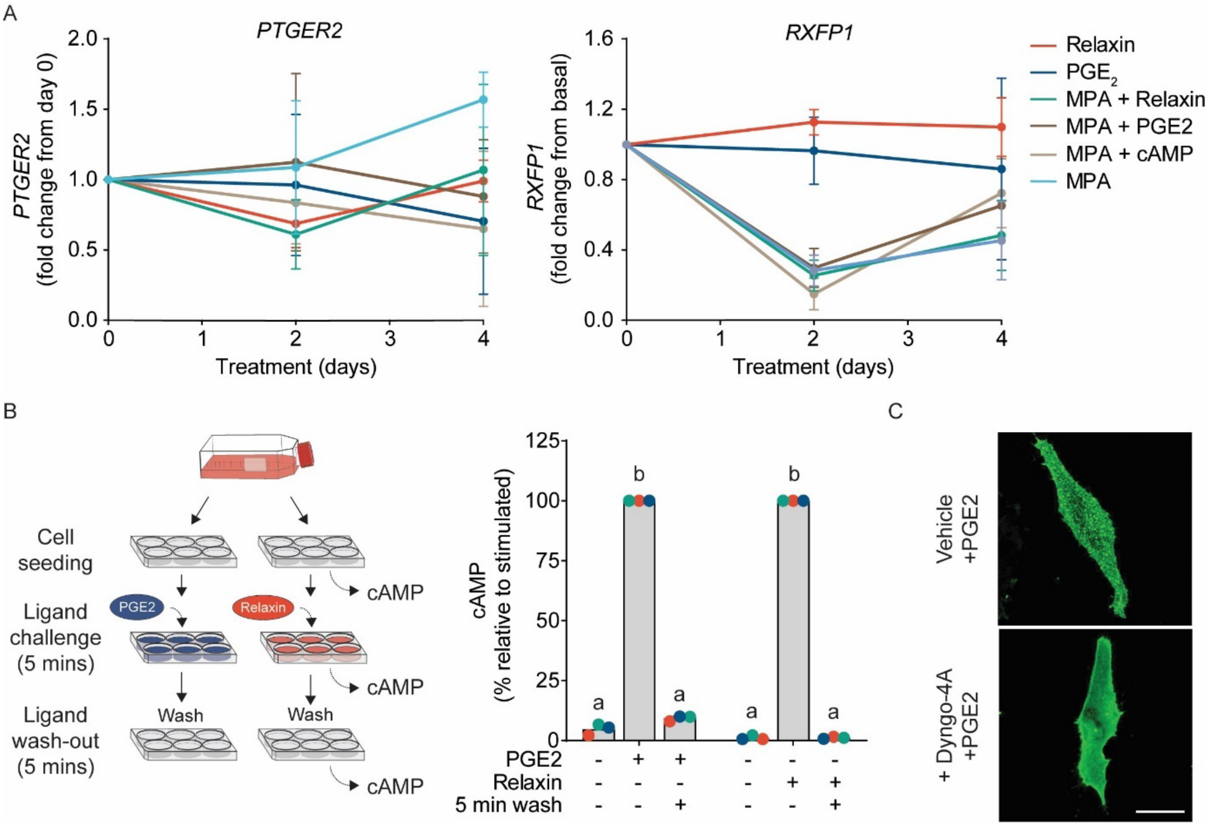

Figure S2. Factors influencing the expression of *PTGER2* and *RXFP1* transcripts, and the regulation of the EP2 and RXFP1 receptors they respectively encode, related to Figure 2. (A) RTqPCR analysis of transcripts coding EP2 (*PTGER2*) (left panel) and RXFP1 (*RXFP1*) (right panel) receptors following various treatments for 2 or 4 days. Data are mean ± SD, n=3. (B) Schematic representation of the experimental procedures used to assess ligand wash out (left panel). Induction of cAMP in endometrial stromal cells following 5-minute ligand wash-out (right panel). Data are shown as individual biological replicates from independent primary cultures with bars denoting mean values. Different letters indicate statistical difference ($p < 0.05$) from treated cells (ANOVA and Šidák's multiple comparison test), n=3. (C) Confocal images of FLAG-Tagged EP2 receptors in cultured EnSCs pre-treated with or without the dynamin inhibitor Dyngo-4A to prevent receptor internalization. Images are representative of 3 further cultures. Scale bar = 20µm.

Figure S3

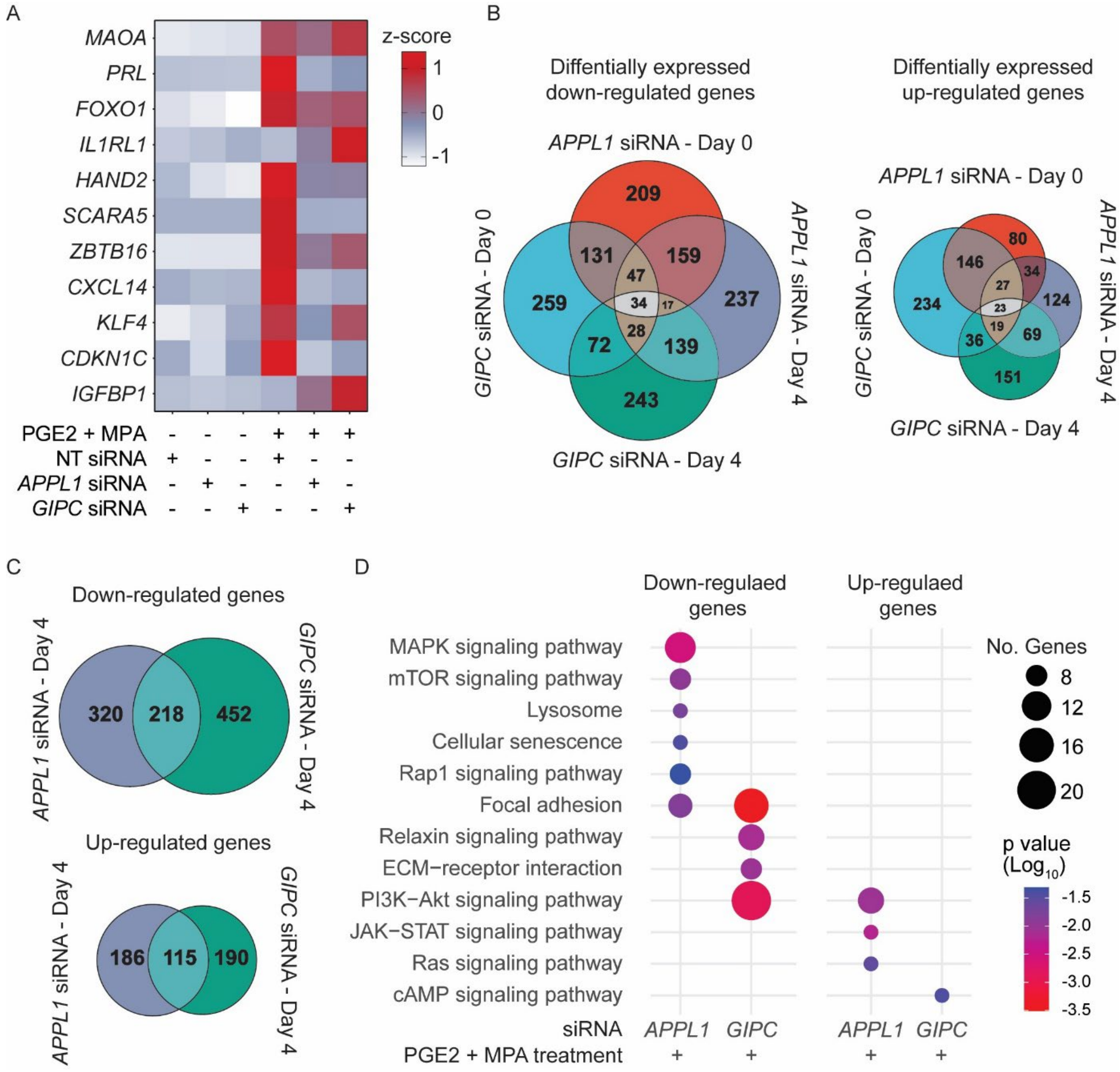

Figure S3. Differentially expressed genes identified after APPL1 and GIPC depletion in EnSCs, related to Figure 5. (A) Heatmap showing relative expression (z-score) of key decidual genes following depletion of APPL1 or GIPC. Red, blue and white represent high, medium and low gene expression, respectively, as indicated by the color key. (B) Venn diagram depicting the number of individual and common differentially genes (Bonferroni correction, $p < 0.05$) identified comparing APPL1 and GIPC depleted EnSCs to treatment-matched NT siRNA-transfected cells. Diagrams are proportional to represent down-regulated genes (left

panel) and up-regulated genes (right panel). (C) Venn diagram showing the number of unique and shared differentially genes after 4 days PGE2/MPA treatment in cells depleted of APPL1 or GIPC. Diagrams are proportional to represent down-regulated genes (upper panel) and up-regulated genes (lower panel). (D) Selected Kyoto Encyclopedia of Genes and Genomes (KEGG) pathway enrichment of up- and down-regulated differentially expressed genes that are exclusive to APPL1 or GIPC depleted EnSC after 4 days treatment with PGE2/MPA. The size of circles is relative to the number of genes in each enrichment term, and the color represents *p* value calculated as a result of enrichment degree.

Table S1. Patient demographics for endometrial biopsies used for EnSC cultures, related to Figures 1-5.

Figure	n	Age	BMI	LH+
Total	46	36.5 (34-39.75)	24 (22-27)	8 (7-10)
1B, 1C	3	37 (36-37.5)	28 (25-28.9)	7 (7-9)
1G, 1H, S1B, S1C	3	40 (37.5-40.5)	24 (22-28)	8 (8-9)
2A	3	32 (31.5-34)	27 (26-28)	7 (7-7)
2C	3	38 (36-41)	22.4 (21.2-24.1)	7 (7-9.5)
2D, S2B	3	30 (19.5-36)	23 (21.5-27)	9 (8.5-10)
2E	3	36 (34.5-38)	23 (22-23.5)	9 (8-9.5)
3B	4	40 (37-41)	23 (22.5-24.95)	8 (7-9)
3C	3	36 (33.5-38)	22 (21.5-28.5)	9 (7.5-10)
S2C	6	38 (33.5-39)	27 (26-28)	8 (7-8.5)
4A, 4B	3	36 (32-37)	25.8 (24-25.9)	9 (7-8)
4C	3	43 (38.5-43)	27 (23.5-31.1)	10 (10-11)
5B-F, S3	3	34 (32.5-36.5)	26 (25-28)	7 (6.5-8)
2G	3	39 (37-39)	20 (19.5-21)	8 (8-9)
Supple 2A	3	38 (34-39)	22 (21.5-23)	7 (6.5-8)

All data are median (interquartile range, Q1-Q3).
LH+: Days since the pre-ovulatory Luteinizing Hormone (LH) surge

Table S2. Full list of KEGG terms from differentially expressed genes comparing *APPL1* and NT siRNA-transfected EnSCs treated with PGE2 + MPA for 4 days, related to Figure 5.

NT siRNA Day 4 vs *APPL1* siRNA Day 4				
**GO Term (KEGG Pathway)**	**No. of genes**	**%**	**Fold Enrichment**	***p* value**
Focal adhesion	31	3.2	2.8	5.80E-07
PI3K-Akt signaling pathway	42	4.4	2.2	3.90E-06
Pathways in cancer	55	5.7	1.9	6.20E-06
Proteoglycans in cancer	28	2.9	2.5	1.90E-05
Protein digestion and absorption	18	1.9	3.2	4.00E-05
Small cell lung cancer	16	1.7	3.2	1.30E-04
ECM-receptor interaction	15	1.6	3.1	3.20E-04
Human papillomavirus infection	33	3.4	1.8	1.20E-03
cAMP signaling pathway	24	2.5	1.9	2.90E-03
Arrhythmogenic right ventricular cardiomyopathy	12	1.3	2.8	3.00E-03
Hypertrophic cardiomyopathy	13	1.4	2.6	3.60E-03
Steroid biosynthesis	6	0.6	5.4	3.80E-03
Dilated cardiomyopathy	13	1.4	2.5	6.10E-03
Toxoplasmosis	14	1.5	2.3	8.20E-03
AGE-RAGE signaling pathway in diabetic complications	13	1.4	2.4	8.40E-03
Amoebiasis	13	1.4	2.3	9.80E-03
Hepatocellular carcinoma	18	1.9	1.9	1.10E-02
EGFR tyrosine kinase inhibitor resistance	11	1.1	2.5	1.10E-02
Relaxin signaling pathway	15	1.6	2.1	1.10E-02
Rap1 signaling pathway	21	2.2	1.8	1.10E-02
MAPK signaling pathway	27	2.8	1.6	1.50E-02
Platinum drug resistance	10	1	2.5	1.80E-02
Complement and coagulation cascades	11	1.1	2.3	1.90E-02
JAK-STAT signaling pathway	17	1.8	1.9	2.00E-02
Fluid shear stress and atherosclerosis	15	1.6	2	2.00E-02
Calcium signaling pathway	23	2.4	1.7	2.20E-02
Axon guidance	18	1.9	1.8	2.20E-02
Pancreatic cancer	10	1	2.4	2.30E-02
Chagas disease	12	1.3	2.1	2.40E-02
Glycosaminoglycan	5	0.5	4.3	2.60E-02
Growth hormone synthesis, secretion and action	13	1.4	2	3.20E-02
Ras signaling pathway	21	2.2	1.6	3.50E-02
Platelet activation	13	1.4	1.9	3.90E-02
Circadian entrainment	11	1.1	2.1	4.00E-02
Other types of O-glycan biosynthesis	7	0.7	2.7	4.20E-02
ErbB signaling pathway	10	1	2.1	4.30E-02
Melanoma	9	0.9	2.3	4.30E-02
Oxytocin signaling pathway	15	1.6	1.8	4.30E-02
Cushing syndrome	15	1.6	1.8	4.50E-02

Table S3. Full list of KEGG terms from differentially expressed genes comparing *GIPC* and NT siRNA-transfected EnSCs treated with PGE2 + MPA for 4 days, related to Figure 5.

NT siRNA Day 4 vs *GIPC* siRNA Day 4				
**GO Term (KEGG Pathway)**	**No. of genes**	**%**	**Fold Enrichment**	***p* value**
cAMP signaling pathway	26	3.1	2.6	2.40E-05
Arrhythmogenic right ventricular cardiomyopathy	14	1.7	4	3.30E-05
Parathyroid hormone synthesis, secretion and action	16	1.9	3.4	7.10E-05
Focal adhesion	23	2.8	2.5	1.10E-04
Cushing syndrome	19	2.3	2.7	1.90E-04
Vascular smooth muscle contraction	17	2.1	2.8	3.10E-04
Dilated cardiomyopathy	14	1.7	3.2	3.30E-04
MAPK signaling pathway	28	3.4	2.1	4.70E-04
cGMP-PKG signaling pathway	19	2.3	2.5	4.80E-04
Adrenergic signaling in cardiomyocytes	18	2.2	2.6	5.20E-04
Oxytocin signaling pathway	18	2.2	2.6	5.20E-04
Cortisol synthesis and secretion	11	1.3	3.8	5.80E-04
Calcium signaling pathway	24	2.9	2.1	9.80E-04
Axon guidance	19	2.3	2.3	1.30E-03
Regulation of actin cytoskeleton	22	2.7	2.1	1.40E-03
Hypertrophic cardiomyopathy	12	1.5	3	2.20E-03
Long-term potentiation	10	1.2	3.3	2.90E-03
mTOR signaling pathway	16	1.9	2.3	4.30E-03
Toxoplasmosis	13	1.6	2.6	4.30E-03
Proteoglycans in cancer	19	2.3	2.1	4.90E-03
TNF signaling pathway	13	1.6	2.5	5.00E-03
Rap1 signaling pathway	19	2.3	2	6.30E-03
Cholesterol metabolism	8	1	3.5	7.30E-03
Pathways in cancer	37	4.5	1.5	8.20E-03
Wnt signaling pathway	16	1.9	2.1	9.80E-03
Aldosterone synthesis and secretion	11	1.3	2.5	1.20E-02
Human papillomavirus infection	25	3	1.7	1.40E-02
Shigellosis	20	2.4	1.8	1.50E-02
Long-term depression	8	1	3	1.70E-02
Growth hormone synthesis, secretion and action	12	1.5	2.2	1.90E-02
Gastric acid secretion	9	1.1	2.6	2.00E-02
Adherens junction	10	1.2	2.4	2.30E-02
Pathways of neurodegeneration - multiple diseases	32	3.9	1.5	2.30E-02
Human cytomegalovirus infection	18	2.2	1.8	2.50E-02
PI3K-Akt signaling pathway	25	3	1.6	2.80E-02
Human immunodeficiency virus 1 infection	17	2.1	1.8	2.90E-02
Circadian entrainment	10	1.2	2.3	3.00E-02
Glutamatergic synapse	11	1.3	2.1	3.40E-02
Serotonergic synapse	11	1.3	2.1	3.40E-02
Melanogenesis	10	1.2	2.2	3.70E-02
Hepatocellular carcinoma	14	1.7	1.9	3.90E-02
Progesterone-mediated oocyte maturation	10	1.2	2.2	3.90E-02
Cardiac muscle contraction	9	1.1	2.3	4.10E-02
Human T-cell leukemia virus 1 infection	17	2.1	1.7	4.20E-02
ECM-receptor interaction	9	1.1	2.2	4.60E-02
Estrogen signaling pathway	12	1.5	1.9	4.60E-02
Apelin signaling pathway	12	1.5	1.9	4.80E-02

Table S4. Full list of KEGG terms from differentially expressed genes comparing unstimulated (Day 0) *APPL1* and NT siRNA-transfected EnSCs, related to Figure 5.

NT siRNA Day 0 vs *APPL1* siRNA Day 0				
**GO Term (KEGG Pathway)**	**No. of genes**	**%**	**Fold Enrichment**	***p* value**
Focal Adhesion	21	2.3	2.1	2.40E-03
Complement and coagulation cascades	12	1.3	2.8	3.20E-03
Protein digestion and absorption	13	1.4	2.6	4.60E-03
Insulin resistance	13	1.4	2.4	6.70E-03
Human immunodeficiency virus 1 infection	20	2.2	1.9	8.30E-03
Regulation of actin cytoskeleton	21	2.3	1.9	9.10E-03
TNF signaling pathway	13	1.4	2.3	1.00E-02
Relaxin signaling pathway	14	1.5	2.2	1.10E-02
Proteoglycans in cancer	19	2.1	1.9	1.20E-02
Arrhythmogenic right ventricular cardiomyopathy	10	1.1	2.6	1.30E-02
Hypertrophic cardiomyopathy	11	1.2	2.5	1.30E-02
Growth hormone synthesis, secretion and action	13	1.4	2.2	1.50E-02
Human cytomegalovirus infection	20	2.2	1.8	1.50E-02
Yersinia infection	14	1.5	2.1	1.70E-02
Fluid shear stress and atherosclerosis	14	1.5	2	1.90E-02
Circadian entrainment	11	1.2	2.3	2.10E-02
Epithelial cell signaling in Helicobacter pylori infection	9	1	2.6	2.10E-02
Human papillomavirus infection	26	2.9	1.6	2.20E-02
Hippo signaling pathway	15	1.7	1.9	2.30E-02
Type II diabetes mellitus	7	0.8	3.1	2.40E-02
AGE-RAGE signaling pathway in diabetic complications	11	1.2	2.2	2.50E-02
MAPK signaling pathway	24	2.6	1.6	2.50E-02
PI3K-Akt signaling pathway	27	3	1.5	2.70E-02
Chagas disease	11	1.2	2.2	2.80E-02
Hepatitis B	15	1.7	1.9	2.90E-02
Valine, leucine and isoleucine degradation	7	0.8	3	2.90E-02
Rap1 signaling pathway	18	2	1.7	3.00E-02
Longevity regulating pathway	10	1.1	2.3	3.10E-02
Chronic myeloid leukemia	9	1	2.4	3.30E-02
Axon guidance	16	1.8	1.8	3.50E-02
cGMP-PKG signaling pathway	15	1.7	1.8	3.60E-02
Insulin signaling pathway	13	1.4	1.9	3.70E-02
Phagosome	14	1.5	1.9	3.70E-02
Salmonella infection	20	2.2	1.6	3.80E-02
Oxytocin signaling pathway	14	1.5	1.8	4.00E-02
TGF-beta signaling pathway	11	1.2	2.1	4.00E-02
Endocytosis	20	2.2	1.6	4.10E-02
Pathways of neurodegeneration - multiple diseases	33	3.6	1.4	4.40E-02
Autophagy - animal	13	1.4	1.9	4.50E-02
N-Glycan biosynthesis	7	0.8	2.7	4.50E-02
Dilated cardiomyopathy	10	1.1	2.1	4.60E-02
Human T-cell leukemia virus 1 infection	18	2	1.6	4.70E-02
Protein processing in endoplasmic reticulum	15	1.7	1.7	4.80E-02
Toxoplasmosis	11	1.2	2	4.90E-02

Table S5. Full list of KEGG terms from differentially expressed genes comparing unstimulated (Day 0) *GIPC* and NT siRNA-transfected EnSCs, related to Figure 5.

NT siRNA Day 0 vs *GIPC* siRNA Day 0				
**GO Term (KEGG Pathway)**	**No. of genes**	**%**	**Fold Enrichment**	***p* value**
Proteoglycans in cancer	30	2.9	2.5	1.00E-05
Vascular smooth muscle contraction	21	2	2.6	1.10E-04
Wnt signaling pathway	23	2.2	2.3	4.70E-04
TGF-beta signaling pathway	17	1.6	2.6	5.80E-04
Pathways in cancer	51	4.9	1.6	6.70E-04
Arrhythmogenic right ventricular cardiomyopathy	13	1.2	2.8	1.80E-03
Hippo signaling pathway	20	1.9	2.1	2.30E-03
IL-17 signaling pathway	14	1.3	2.5	3.50E-03
Spinocerebellar ataxia	18	1.7	2.1	4.60E-03
Breast cancer	18	1.7	2.1	6.10E-03
Hypertrophic cardiomyopathy	13	1.2	2.4	6.60E-03
MAPK signaling pathway	30	2.9	1.7	6.80E-03
Renin secretion	11	1	2.7	7.10E-03
NF-kappa B signaling pathway	14	1.3	2.3	8.30E-03
Focal adhesion	22	2.1	1.8	8.90E-03
Regulation of actin cytoskeleton	24	2.3	1.8	9.00E-03
Shigellosis	25	2.4	1.7	1.10E-02
NOD-like receptor signaling pathway	20	1.9	1.8	1.40E-02
Calcium signaling pathway	25	2.4	1.7	1.50E-02
Gap junction	12	1.1	2.3	1.50E-02
Yersinia infection	16	1.5	2	1.50E-02
Estrogen signaling pathway	16	1.5	2	1.60E-02
TNF signaling pathway	14	1.3	2.1	1.70E-02
Human papillomavirus infection	30	2.9	1.5	2.20E-02
Pathogenic Escherichia coli infection	20	1.9	1.7	2.40E-02
Dilated cardiomyopathy	12	1.1	2.1	2.60E-02
Sphingolipid signaling pathway	14	1.3	1.9	2.70E-02
Pertussis	10	1	2.2	3.50E-02
Pathways of neurodegeneration - multiple diseases	39	3.7	1.4	3.60E-02
Sphingolipid metabolism	8	0.8	2.5	3.60E-02
JAK-STAT signaling pathway	17	1.6	1.7	3.70E-02
Leishmaniasis	10	1	2.2	3.80E-02
cGMP-PKG signaling pathway	17	1.6	1.7	3.90E-02
Salmonella infection	23	2.2	1.6	3.90E-02
Axon guidance	18	1.7	1.7	4.20E-02
Rap1 signaling pathway	20	1.9	1.6	4.30E-02
Tight junction	17	1.6	1.7	4.30E-02
Cytokine-cytokine receptor interaction	26	2.5	1.5	4.80E-02
